# Microbial Distribution and Antibiotic Susceptibility of Bloodstream Infections in Different Intensive Care Units

**DOI:** 10.3389/fmicb.2021.792282

**Published:** 2021-12-09

**Authors:** Nan Duan, Liying Sun, Chenwei Huang, Haixia Li, Bin Cheng

**Affiliations:** ^1^Department of Clinical Laboratory, Peking University First Hospital, Beijing, China; ^2^Department of Clinical Laboratory, Miyun Teaching Hospital, Capital Medical University, Beijing, China

**Keywords:** bloodstream infections, antibiotic susceptibility, *Staphylococcus hominis*, *Klebsiella pneumoniae*, microbial distributions

## Abstract

**Background:** Bloodstream infection (BSI) is an increasing public health concern worldwide, representing a serious infection with significant morbidity and mortality, especially in children and the elderly. The predominant microbial distribution and antibiotic susceptibility were investigated among BSIs in the different intensive care units (ICUs)—pediatric ICU (PICU), surgical ICU (SICU), cardiac ICU (CICU), respiratory ICU (RICU), and geriatric ICU (GICU)—in order to achieve more efficient and appropriate therapies for patients in various ICUs.

**Methods:** In this retrospective cross-sectional study, the blood specimens were collected from five different ICUs of Peking University First Hospital and comprehensive ICU of Miyun Teaching Hospital (Miyun ICU) before antimicrobial treatment from 2017 to 2020. Microorganism cultures of the blood samples were conducted, and positive cultures were tested for type of pathogens and drug susceptibility.

**Results:** The prevalence of BSIs was the highest in the Miyun ICU (10.85%), followed by the RICU (9.48%) and the PICU (8.36%). The total prevalence of Gram-positive bacterial strains (especially *Staphylococcus spp.* and *Enterococcus spp.*) in the PICU (44.55%), SICU (57.58%), CICU (55.00%), GICU (49.06%), and Miyun ICU (57.58%) was higher than that of Gram-negative bacteria. The major bacterial strain was *Acinetobacter baumannii* in the PICU (21.82%); *Klebsiella pneumoniae* in the SICU (12.88%), CICU (30.00%), and RICU (30.39%); *Escherichia coli* in the GICU (20.75%); and *Staphylococcus epidermidis* (18.18%) in the Miyun ICU. *Staphylococcus hominis* of BSIs remained highly susceptible (>70%) to gentamicin, linezolid, daptomycin, teicoplanin, vancomycin, tigecycline, and rifampicin in all the ICUs. Its antibiotic sensitivity to levofloxacin was moderate in the PICU and CICU, but mild (<30%) in the SICU, RICU, and GICU. *K*. *pneumoniae* was highly susceptible to doxycycline, minocycline, and tigecycline in all the ICUs except the RICU, and its antibiotic sensitivity to imipenem, meropenem, amikacin, ciprofloxacin, and levofloxacin was high/moderate in the PICU, CICU, GICU, and Miyun ICU, but mild in the SICU and RICU.

**Conclusion:** The current study demonstrated the distribution of prevalent microorganisms, and their antimicrobial susceptibility exhibited a high divergence among BSIs in different ICUs from a tertiary hospital and an outer suburban hospital in Beijing. Therefore, different antibiotic therapies for various wards and distinct age groups (especially between pediatric and elderly patients) should be considered to control the emergence and spread of highly antibiotic-resistant infections.

## Background

Bloodstream infection (BSI), a serious infection with significant morbidity and mortality, becomes an increasing public health concern worldwide. Population-based studies in North America and Europe demonstrate that the BSI incidence ranged between 113 and 204 per 100,000 population (Goto and Al-Hasan, 2013; Kern and Rieg, 2020). A previous research based on the Swiss antibiotic resistance surveillance system (ANRESIS) described temporal trends and revealed a rise in the incidence rate of BSIs (increased by 14%, from 211 in 2008 to 240 per 100,000 population in 2014), especially among elderly patients (≥65 years) (Buetti et al., 2017). Moreover, it is also reported that BSIs remained one of the leading causes of death with an overall estimated fatality rate of 12.7% in the pediatric population (Droz et al., 2019). Antimicrobial agents are commonly administered to treat BSIs, but the clinical management is not so effective because of antibiotic resistance.

In recent years, the rise in multidrug-resistant pathogens is one of the most serious global public health threats, and the World Health Organization (WHO) has published the first global report on surveillance of antimicrobial resistance, showing the extent of this phenomenon in many parts of the world (World Health Organization., 2014). Empirical therapeutics is often started immediately in suspected BSI to reduce associated morbidity and mortality because of the routine delays in receiving blood culture and antibiotic susceptibility analysis. However, the current international guidelines are not suitable for all regions and generic recommended antimicrobial therapy is sometimes not adapted to local susceptibility patterns in developing countries (Penno et al., 2015; Labi et al., 2016). Changing antimicrobial resistance rates, pathogen distribution, demographics, and medical care delivery all may affect the epidemiology of BSI. Therefore, updated trends in the microbiology of BSI pathogens and the antibiotic susceptibility are required to support therapeutic guidelines for appropriate therapies.

The effective selection and use of antibiotics lately has become a huge challenge for all physicians. However, a comparison of relative frequency and microbiological characteristics of the pathogens in patients with BSIs in different intensive care units (ICU) was seldom investigated thoroughly in China, particularly in elderly (>60 years) and pediatric patients. In the this study, the microbial distribution and antibiotic sensitivity of BSIs in the pediatric ICU (PICU), surgical ICU (SICU), cardiac ICU (CICU), respiratory ICU (RICU), and geriatric ICU (GICU) from a tertiary hospital and the comprehensive ICU from an outer suburban hospital were investigated to achieve more efficient treatment protocols and better recovery in the hospital.

## Materials and Methods

### Subjects

We conducted a cross-sectional study with a retrospective cohort in the Department of PICU, Department of SICU, Department of CICU, Department of RICU, and Department of GICU in Peking University First Hospital (PKUFH, a large-scale tertiary hospital in Beijing), and Department of Comprehensive ICU in Miyun Teaching Hospital (a small-scale hospital located on the outer suburb of Beijing) (Miyun ICU) from January 1, 2017, to June 30, 2020. Five hundred sixteen positive culture samples from 6,597 blood samples in the PICU, SICU, CICU, RICU, and GICU of PKUFH and 275 positive cultures from 2,534 blood specimens in the Miyun ICU were analyzed (Table 1). The blood samples were collected before antimicrobial treatment. BSIs are defined by the presence of an organism within the bloodstream with or without signs or symptoms of an infection (Watson and Al-Hasan, 2014). The clinical clues were also taken into account for BSI validation; for example, the number of white blood cells, value of C-reactive protein and procalcitonin, and clinical signs of infection such as a fever and chills. This study has been reviewed and approved by the Institutional Ethics Committee of Peking University First Hospital and Miyun Teaching Hospital, and a review in terms of patient consent was not needed because of the retrospective observational design according to the committee decision.

**TABLE 1:** Blood cultures of patients in different intensive care units.

	PICU	SICU	CICU	RICU	GICU	Miyun ICU
Male (%)	51.67	61.11	55.45	60.53	70.34	69.85
Age (years)	4.69 ± 1.61	58.78 ± 11.52	68.57 ± 16.54	68.43 ± 16.53	84.33 ± 9.72	66.44 ± 14.74
Blood cultures						
Total samples (N)	1316	2160	541	1909	671	2534
Positive isolates (n)	110	132	40	181	53	275
Prevalence (%)	8.36	6.11	7.39	9.48	7.90	10.85
Resistance patterns (n/%)						
ESBL	1/0.91	3/2.27	0/0.00	0/0.00	4/7.55	14/5.09
CRE	4/3.64	17/12.88	5/12.5	49/27.07	3/5.66	19/6.91
CRAB	21/19.09	4/3.03	0/0.00	9/4.97	1/1.88	4/1.45
MRSA	0/0.00	1/0.76	0/0.00	4/2.21	0/0.00	0/0
MRCNS	24/21.82	36/27.27	10/25.00	49/27.07	11/20.75	99/36.00
VRE	0/0.00	0/0.00	1/2.5	2/1.10	0/0.00	2/0.73

*Continuous values are expressed as mean ± SD. PICU, pediatric intensive care unit; SICU, surgical intensive care unit; CICU, cardiac intensive care unit; RICU, respiratory intensive care unit; GICU, geriatric intensive care unit; ESBL, extended-spectrum β-lactamase; CRE, carbapenem-resistant Enterobacteriaceae; CRAB, carbapenem-resistant Acinetobacter baumannii; MRSA, methicillin-resistant Staphylococcus aureus; MRCNS, methicillin-resistant coagulase-negative Staphylococcus; VRE, vancomycin-resistant Enterococcus.*

### Microorganism Identification and Drug-Susceptibility Test

Microorganism cultures were conducted according to the routine standard operation procedure (SOP) described previously (Duan et al., 2020): blood specimen was inoculated into a commercial culture bottle and analyzed by an automated monitoring system for bacterial detection (BD BACTEC™, BD, San Jose, CA, United States). The incubation was continued until a positive culture was observed or up to a maximum of 5 days. Positive bacterial cultures from patients’ blood samples collected within the first 24 h of admission were tested for type of pathogens and sensitivity to antibiotics. The microorganisms were identified to species level by standard biochemical methods including matrix-assisted laser desorption ionization-time of flight mass spectrometry (MALDI-TOF MS) (Bruker Daltonics, Billerica, MA, United States) and automated methods using the VITEK system (bioMérieux, Marcy-l’Étoile, France). The bacterial isolates were tested for antimicrobial susceptibility by VITEK 2 Compact (bioMérieux, France) or the manual Kirby–Bauer (K–B) disk diffusion method. Interpretations of drug susceptibility data were based on the Clinical Laboratory Standards Institute (CLSI, United States) standard.

### Statistical Analysis

Continuous values of ages are expressed as mean ± SD. Microbial distribution and antibiotic susceptibility data were presented as percentages. Descriptive statistical analysis of the quantile–quantile plot (Q–Q plot) was used to determine the normality of demographic data of patient, and the chi-square (χ^2^) test was performed to compare differences in microbial distribution among different ICUs using SPSS version 16.0 software (SPSS Inc., Chicago, IL, United States). A *p* < 0.05 was considered statistically significant.

## Results

### Prevalence of Positive Blood Cultures in Different Intensive Care Units

**FIGURE 1:**
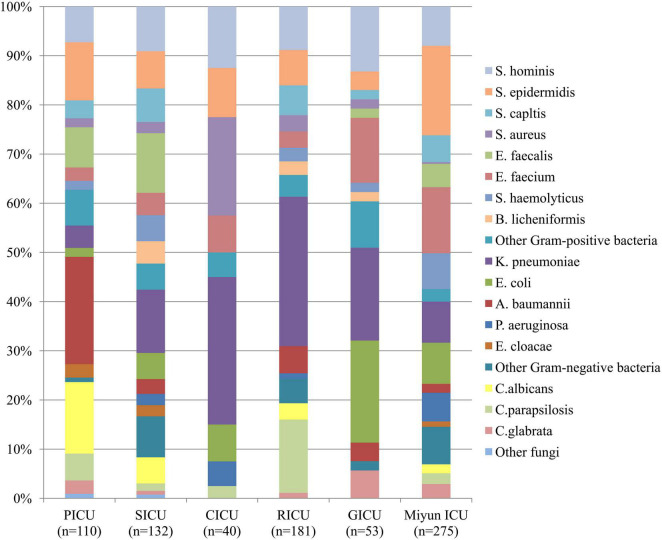
Distribution of microorganisms of bloodstream infections patients from different intensive care units (ICU). Gram-positive bacteria remained the major pathogens among the blood cultures except the respiratory intensive care unit (RICU).

**FIGURE 2:**
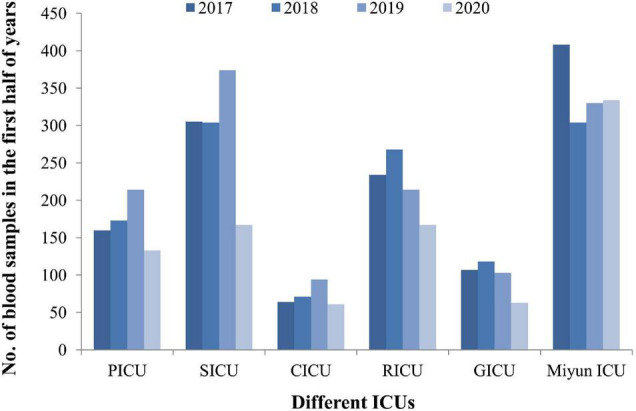
The blood sample collection covers the first half of 2020 when COVID-19 emerged and spread during this period. The lowest amount of blood samples was collected in the five ICUs of PKUFH in 2020, relative to the first half of the other 3 years. The amount of blood specimens of Miyun ICU did not change much in 2020.

From January 1, 2017, to June 30, 2020, the frequencies of reportedly positive cultures were 110 out of 1,316 blood samples in the PICU; 132 out of 2,160 in the SICU; 40 out of 541 in the CICU; 181 out of 1,909 in the RICU; 53 out of 671 in the GICU; and 275 out of 2,534 in the Miyun ICU (Figure 1). The blood sample collection covers the first half of 2020 when COVID-19 emerged and spread. The number of patients or hospital admission rates might be reflected on the changes in the amounts of samples sent to our laboratories. It was found that the lowest amount of blood samples was collected in the five ICUs of PKUFH in 2020, relative to the first half of the other 3 years (Figure 2). However, the amount of blood specimens of Miyun ICU did not change much. The prevalence of positive blood cultures of the patients in different ICUs is summarized in Table 1. The prevalence of positive blood cultures of Miyun ICU was significantly higher than that in five ICUs of PKUFH (10.85% vs. 7.82%, *p* < 0.05). Among the five ICUs of PKUFH, the RICU had the highest prevalence of BSIs (9.48%), followed by the PICU (8.36%), and SICU had the lowest one (6.11%) (*p* < 0.05). The major resistance patterns of bacterial pathogens were methicillin-resistant coagulase-negative *Staphylococcus* (MRCNS) (21.82%) and carbapenem-resistant *Acinetobacter baumannii* (CRAB) (19.09%) in the PICU; MRCNS in the SICU (27.27%), CICU (25.00%), GICU (20.75%), and Miyun ICU (36.00%); and MRCNS (27.07%) and carbapenem-resistant *Enterobacteriaceae* (CRE) (27.07%) in the RICU.

### Distribution and Comparison of Microorganisms in Blood Cultures

The profile of microorganisms isolated from BSI patients is summarized in Figure 1. In the PICU, the most common bacterial strains in blood samples were *Acinetobacter baumannii* (*A*. *baumannii*) (*n* = 24, 21.82%) and *Staphylococcus epidermidis* (*S*. *epidermidis*) (*n* = 13, 11.82%), followed by *Enterococcus faecalis* (*E*. *faecalis*) (*n* = 9, 8.18%) and *Staphylococcus hominis* (*S*. *hominis*) (*n* = 8, 7.27%). In the SICU, the four most frequent pathogens were *Klebsiella pneumoniae* (*K*. *pneumoniae*) (*n* = 17, 12.88%), *E. faecalis* (*n* = 16, 12.12%), *S*. *hominis* (*n* = 12, 9.09%), and *S*. *epidermidis* (*n* = 10, 7.58%) among blood cultures. Gram-positive bacteria (*n* = 22, 55.00%) accounted for over 50% in blood specimens of patients admitted to the CICU, but Gram-negative *K*. *pneumoniae* (*n* = 12, 30.00%) was the dominant microorganism. *Staphylococcus aureus* (*S*. *aureus*) (*n* = 8, 20.00%) was the second most common bacterium, followed by *S*. *hominis* (*n* = 5, 12.50%) and *S. epidermidis* (*n* = 4, 10.00%). In the RICU, Gram-negative bacteria (*n* = 76, 41.99%) remained the major pathogens of patients’ blood cultures, and the five most frequent bacterial strains were *K*. *pneumoniae* (*n* = 55, 30.39%), *S*. *hominis* (*n* = 16, 8.84%), *S. epidermidis* (*n* = 13, 7.18%), *Staphylococcus capitis* (*S*. *capitis*) (*n* = 11, 6.08%), and *A. baumannii* (*n* = 10, 5.52%). The most predominant microorganism in the GICU was *Escherichia coli* (*E*. *coli*) (*n* = 11) with an isolation rate of 20.75%, followed by *K*. *pneumoniae* (*n* = 10, 18.87%), *S*. *hominis* (*n* = 7, 13.21%), and *Enterococcus faecium* (*E*. *faecium*) (*n* = 7, 13.21%). In the Miyun ICU, the most common bacterial strains in blood samples were *S. epidermidis* (*n* = 50, 18.18%), followed by *E*. *faecium* (*n* = 37, 13.45%), *K*. *pneumoniae* (*n* = 23, 8.36%), *E*. *coli* (*n* = 23, 8.36%), and *S. hominis* (*n* = 22, 8.00%).

Among the blood cultures, the frequency of Gram-positive bacterial strains (especially *Staphylococcus spp.* and *Enterococcus spp.*) was higher than that of Gram-negative bacterial strains in the PICU (*n* = 49, 44.55%), SICU (*n* = 76, 57.58%), CICU (*n* = 22, 55.00%), GICU (*n* = 26, 49.06%), and Miyun ICU (*n* = 76, 57.58%), of which, however, Gram-negative *A. baumannii*, *K*. *pneumoniae*, and *E. coli* were the most prevalent. In addition, *K*. *pneumoniae* was the dominant pathogen in three of the five ICUs of PKUFH (such as the SICU, CICU, and RICU). Fungal pathogens in the PICU, SICU, CICU, RICU, GICU, and Miyun ICU were, respectively, at 23.64, 8.33, 2.50, 19.34, 5.66, and 6.91%, and *Candida albicans* and *Candida parapsilosis* were the most common fungi.

### Antibiotic Susceptibility of Major Bacteria of Bloodstream Infections

**FIGURE 3:**
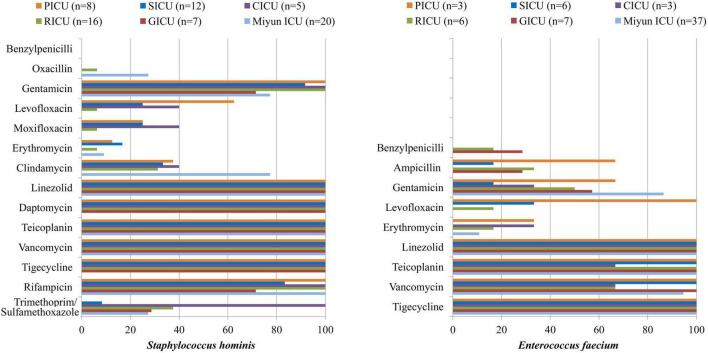
Major Gram-positive bacteria isolated and their drug-susceptibility rates in patients with bloodstream infections from different intensive care units (ICU). The antimicrobial susceptibility performed by VITEK 2 Compact or the manual Kirby–Bauer (K–B) disk diffusion method.

The drug susceptibility of Gram-positive *S. hominis* and *E. faecium* was selected, as shown in Table 2 and Figure 3. As the predominant Gram-positive bacterium, *S. hominis* remained highly susceptible (>70%) to gentamicin, linezolid, daptomycin, teicoplanin, vancomycin, tigecycline, and rifampicin in all the ICUs, moderately susceptible (30–70%) to clindamycin apart from the GICU. Its antibiotic sensitivity to levofloxacin was moderate in the PICU and CICU, but mild (<30%) in the SICU, RICU, and GICU. *E. faecium* exhibited high sensitivity to linezolid and tigecycline and mild sensitivity to benzylpenicillin and erythromycin in all the ICUs, and it was highly sensitive to teicoplanin and vancomycin in the PICU, SICU, GICU (except RICU and CICU), and Miyun ICU. Its drug susceptibility to ampicillin and gentamicin was moderate in PICU and mild in other ICUs.

**TABLE 2:** Drug-susceptibility of major Gram-positive bacteria of bloodstream infections in different intensive care units (%).

Antibacterials	*Staphylococcus hominis*						*Enterococcus faecium*					
	PICU (*n* = 8)	SICU (*n* = 12)	CICU (*n* = 5)	RICU (*n* = 16)	GICU (*n* = 7)	Miyun ICU (*n* = 22)	PICU (*n* = 3)	SICU (*n* = 6)	CICU (*n* = 3)	RICU (*n* = 6)	GICU (*n* = 7)	Miyun ICU (*n* = 37)
Benzylpenicillin	0	0	0	0	0	–	0	0	0	16.67	28.57	–
Ampicillin	–	–	–	–	–	–	66.67	16.67	0	33.33	28.57	0
Oxacillin	0	0	0	6.25	0	27.27	–	–	–	–	–	–
Gentamicin	100	91.67	100	100	71.43	77.27	66.67	16.67	33.33	50	57.14	86.48
Levofloxacin	62.5	25	40	6.25	0	–	100	33.33	0	16.67	0	–
Moxifloxacin	25	25	40	6.25	0	–	–	–	–	–	–	–
Erythromycin	12.50	16.67	0	6.25	0	9.09	33.33	0	33.33	16.67	0	10.81
Clindamycin	37.5	33.33	40	31.25	0	77.27	–	–	–	–	–	–
Linezolid	100	100	100	100	100	100	100	100	100	100	100	100
Daptomycin	100	100	100	100	100	–	–	–	–	–	–	–
Teicoplanin	100	100	100	100	100	100	100	100	66.67	100	100	100
Vancomycin	100	100	100	100	100	100	100	100	66.67	66.67	100	94.59
Tigecycline	100	100	100	100	100	–	100	100	100	100	100	–
Rifampicin	100	83.33	100	100	71.43	100	–	–	–	–	–	–
Trimethoprim/sulfamethoxazole	0	8.33	100	37.5	28.57	27.27	–	–	–	–	–	–

**FIGURE 4:**
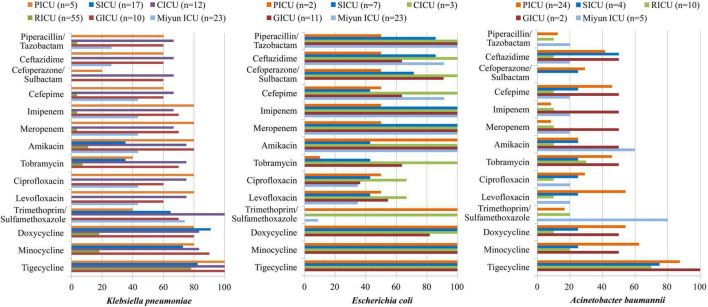
Major Gram-negative bacteria isolated and their drug-susceptibility rates in patients with bloodstream infections from different intensive care units (ICU). The antimicrobial susceptibility performed by VITEK 2 Compact or the manual Kirby–Bauer (K–B) disk diffusion method.

The antibiotic sensitivity of Gram-negative *K*. *pneumoniae*, *E. coli*, and *A. baumannii* was selected, as shown in Table 3 and Figure 4. *K*. *pneumoniae* of BSIs remained highly sensitive to doxycycline, minocycline, and tigecycline in all the ICUs of PKUFH except RICU and moderately susceptible to piperacillin/tazobactam, ceftazidime, cefoperazone/sulbactam, and cefepime in the PICU, CICU, and GICU. Its antibiotic sensitivity to imipenem, meropenem, and amikacin was high in PICU, CICU, and GICU, moderate in the Miyun ICU, and mild in the SICU and RICU. Moreover, the susceptibility to ciprofloxacin and levofloxacin was high in the PICU and CICU, moderate in the GICU and the Miyun ICU, and still mild in the SICU and RICU. *E. coli* was highly susceptible to the tested antibiotics in the CICU and highly susceptible to meropenem, imipenem, ceftazidime, and piperacillin/tazobactam in the SICU, GICU, and Miyun ICU, whereas it exhibited moderate or relatively low drug susceptibility to other antibiotics except doxycycline, minocycline, and tigecycline in the PICU, SICU, and GICU. *A. baumannii* was highly sensitive to tigecycline and mildly sensitive to piperacillin/tazobactam, cefoperazone/sulbactam, imipenem, meropenem, and ciprofloxacin in the ICUs. Moreover, its susceptibility to ceftazidime, cefepime, tobramycin, levofloxacin, doxycycline, and minocycline was moderate in PICU, but mild in the SICU and RICU.

**TABLE 3:** Drug susceptibility of major Gram-negative bacteria of bloodstream infections in different intensive care units (%).

Antibacterials	*Klebsiella pneumoniae*						*Escherichia coli*						*Acinetobacter baumannii*					
	PICU (*n* = 5)	SICU (*n* = 17)	CICU (*n* = 12)	RICU (*n* = 55)	GICU (*n* = 10)	Miyun ICU (*n* = 23)	PICU (*n* = 2)	SICU (*n* = 7)	CICU (*n* = 3)	RICU (*n* = 0)	GICU (*n* = 11)	Miyun ICU (*n* = 23)	PICU (*n* = 24)	SICU (*n* = 4)	CICU (*n* = 0)	RICU (*n* = 10)	GICU (*n* = 2)	Miyun ICU (*n* = 5)
Piperacillin/tazobactam	60.00	0	66.67	3.64	60.00	26.08	50.00	85.71	100	–	100	100	12.5	0	–	10.00	0	20
Ceftazidime	60.00	0	66.67	0	60.00	26.08	50.00	85.71	100	–	63.64	91.3	45.83	50.00	–	10.00	50.00	20
Cefoperazone/sulbactam	20.00	0	66.67	0	60.00	–	50.00	71.43	100	–	90.91	–	29.17	25.00	–	0	0	–
Cefepime	60.00	0	66.67	3.64	60.00	43.47	50.00	42.85	100	–	63.64	91.3	45.83	25.00	–	10.00	50.00	20
Imipenem	80.00	0	66.67	3.64	70.00	43.47	50.00	100	100	–	100	100	8.33	0	–	10.00	50.00	20
Meropenem	80.00	0	66.67	3.64	70.00	43.47	50.00	100	100	–	100	100	8.33	0	–	10.00	50.00	20
Amikacin	80.00	35.29	75.00	10.91	80.00	43.47	100	42.85	100	–	100	100	25.00	25.00	–	10.00	50.00	60
Tobramycin	40.00	35.29	75.00	7.27	70.00	–	10.00	42.85	100	–	63.64	–	45.83	25.00	–	30.00	50.00	–
Ciprofloxacin	80.00	0	75.00	0	60.00	43.47	50.00	42.85	66.67	–	36.36	34.78	29.17	25.00	–	10.00	0	20
Levofloxacin	80.00	0	75.00	0	60.00	43.47	50.00	42.85	66.67	–	54.55	34.78	54.17	25.00	–	10.00	0	20
Trimethoprim/sulfamethoxazole	40.00	64.71	100	0	70.00	73.91	100	0	100	–	0	8.69	16.67	0	–	20.00	0	80
Doxycycline	80.00	90.91	83.33	18.18	80.00	–	100	100	100	–	81.81	–	54.17	25.00	–	10.00	50	–
Minocycline	80.00	72.73	83.33	18.18	90.00	–	100	100	100	–	100	–	62.50	25.00	–	20.00	50	–
Tigecycline	100	82.35	100	78.18	100	–	100	100	100	–	100	–	87.50	75.00	–	70.00	100	–

## Discussion

In the present study, we carried out a retrospective cross-sectional study from January 1, 2017, to June 30, 2020, in the Department of PICU, SICU, CICU, RICU, and GICU in Peking University First Hospital and Department of Comprehensive ICU in Miyun Teaching Hospital to analyze the positive blood cultures isolated from patients with BSIs. It was demonstrated that the microbial distribution and antibiotic susceptibility exhibited a high divergence among BSIs in different ICUs. Therefore, different antibiotic therapies for various wards and distinct age groups were suggested to prevent highly antibiotic-resistant infections, especially in the RICU and GICU.

Bloodstream infections comprise a wide variety of pathogens and clinical syndromes with quite diverse risk factors, therapeutic implications, and outcomes. The infections occur when one or more pathogens invade the bloodstream, usually causing a systemic inflammatory response syndrome (SIRS; Del Bono and Giacobbe, 2016). BSIs were defined as the isolation of pathogenic organisms from at least one blood culture sample. The detection of the causing pathogen by using molecular techniques has been proven suboptimal, and blood culture remains the gold standard and first-line tool in the pathogen diagnostics of BSIs and sepsis (Rhodes et al., 2017). Microbiological cultures of blood specimens provided clinically relevant information concerning the identity and analysis of microorganisms with their susceptibility to antibiotics. The prevalence of positive blood cultures of Miyun Teaching Hospital comprehensive ICU was the highest (10.85%) among all the ICUs, which indicated that the infection control and treatment might need to be improved in this outer suburban hospital. In Peking University First Hospital, we found that blood specimens of the RICU had the highest positive frequency (9.48%), similar to the finding in an Indian tertiary care institute where SIRS-related sepsis and BSI were 11.5% in the RICU (Agarwal et al., 2006). Previous studies observed a percentage of positive blood culture from 7 to 9.89% in children (Deen et al., 2012; Le Doare et al., 2015), and the presence of BSIs was also relatively high (8.36%) in the PICU, probably due to the immature state of children’s immune system predisposing them to infections.

This study was carried out from January 1, 2017, to June 30, 2020, and the blood sample collection covers the first half of 2020 when COVID-19 emerged and spread. The number of patients or hospital admission rates might be reflected on the changes in the amounts of samples sent to our laboratories. Blood samples collected in the five ICUs of PKUFH in 2020 were decreased, relative to the first half of the other 3 years. However, the amount of blood specimens of Miyun ICU did not change much. The possible reason is that many patients from other cities would come to Peking University First Hospital for treatment before the outbreak of COVID-19, but fewer people came from other areas due to the prevention and control of the epidemic thereafter. The patient population of Miyun Teaching Hospital is mainly located in the suburban; thus, the amount of specimens does not change significantly. These two hospitals are not designated as medical institutions for treatment of COVID-19 patients (all of the COVID-19 patients in Beijing were hospitalized to Beijing Ditan Hospital). All the patients admitted are not infectious with SARS-CoV-2, and no nosocomial infection related to COVID-19 happened in the hospitals. Therefore, effects of COVID-19 infection on patients with BSIs are not considered in the present study.

The distribution of microorganisms isolated from blood specimens showed that the total proportions of Gram-positive bacteria in the PICU, SICU, CICU, GICU, and Miyun ICU were all much higher than Gram-negative bacterial strains. Among these positive blood cultures, several frequently isolated pathogens were *S. hominis*, *S. epidermidis*, and *S. capitis* which belong to coagulase-negative staphylococci (CoNS). In recent years, CoNS have become true pathogens, rather than simply culture contaminants, causing BSIs and cardiovascular infections among other conditions (Yamada et al., 2017). Another research also reported a similar finding that CoNS were the common causative agents of BSI in European populations (Huttunen et al., 2015). Moreover, *Enterococcus spp.* (such as *E. faecalis* and *E. faecium*) was one of the prevalent organisms in the PICU, SICU, GICU, and Miyun ICU, and *S. aureus* was the second most frequent bacterium in CICU, which might indicate that skin, soft tissue, bone, and joint sources of infection caused BSI by Gram-positive cocci such as *S. aureus*, enterococci, and streptococci. In the PICU, the major bacterium was Gram-negative *A*. *baumannii*, followed by *S*. *epidermidis* and *E*. *faecalis*. However, Singhi et al. (2008) from India and Maham et al. (2018) from Iran observed Gram-negative bacteria as the predominant isolates of pediatric BSIs, common being *K. pneumoniae* (20.1% and 8.8%) and *Acinetobacter spp.* (8.6% and 7.9%). The difference between our research and theirs may be due to the distinct social, economic, and environmental factors in these regions. *K*. *pneumoniae* was the most frequent pathogen in three of the six ICUs (the SICU, CICU, and RICU), consistent with a recent review showing that *Klebsiella spp.*, the second (behind *E. coli*) overall cause of Gram-negative BSIs, have been and remain one of key pathogens from high-income countries (Kern and Rieg, 2020). In the GICU, the dominant microorganism was *E. coli* which was also reported as the most prevalent pathogen by a previous research based on the Swiss ANRESIS, revealing that the incidence of BSIs increased throughout the study period 2008–2014, especially among geriatric patients (Buetti et al., 2017). In the Miyun ICU, the most common isolates of blood samples were *S. epidermidis* (the frequency was 18.18%, much higher than other ICUs), which might be partly due to sample or culture contaminants, and thus, it is suggested to standardize specimen collection for patients’ blood culture in this suburban hospital. It is worth noting that the RICU was the only ICU where Gram-negative bacteria remained the key microorganisms involved in BSIs because *Enterobacteriaceae spp.* (such as *K*. *pneumoniae*) might be the predominant microorganisms in respiratory tract infection patients (Mahendra et al., 2018; Duan et al., 2020). A 3-year multicenter retrospective study showed that individuals aged 0–5 years and ≥40 years were the main demographics at risk of infection by *E. coli*, *K. pneumoniae*, and *A. baumannii*, while individuals aged 0–5 years were the major demographic at risk of infection by *S. aureus*, *E. faecalis*, *E. faecium*, etc (Tian et al., 2018). According to our study, there was also a high divergence between pediatric and geriatric patients, or among different ICUs.

In the hospital setting, intensive and prolonged use of antimicrobial drugs introduces the emergence and spread of highly antibiotic-resistant infections (Prestinaci et al., 2015). Currently, antimicrobial resistance is one of the most serious global public health threats which call for action from all of society. In order to achieve appropriate therapeutics, an updated epidemiology of antimicrobial sensitivity is required to support therapeutic guidelines. In the present study, we observed that *S. hominis* of BSIs remained highly susceptible (>70%) to gentamicin, linezolid, daptomycin, teicoplanin, vancomycin, tigecycline, and rifampicin in all the ICUs. Its antibiotic sensitivity to levofloxacin was moderate in the PICU and CICU, but mild (<30%) in the SICU, RICU, and GICU. Moreover, MRCNS was one of the major resistance patterns of positive isolates from the ICUs in this study, and a 4-year retrospective survey in a Japanese tertiary hospital revealed that MRCNS bacteremia (diagnosed as two or more positive blood cultures on the same day with clinical signs of infection) was associated with a low mortality rate and suggested that glycopeptides, especially teicoplanin, must be used appropriately to prevent antibiotic resistance in MRCNS (Yamada et al., 2017). It is vital to treat staphylococcus infections as the increasing proportion of CoNS isolates presented as methicillin-resistant. *E. faecium* exhibited high susceptibility to linezolid and tigecycline in all the ICUs, and it was highly susceptible to teicoplanin and vancomycin in the PICU, SICU, and Miyun ICU. A meta-analysis revealed that the prevalence of vancomycin-resistant Enterococcus (VRE) infections in Iran was 9.4% among culture-positive cases for *Enterococcus spp.*, and analyzed the prevalence of multidrug-resistant VRE in developed countries, such as Germany (11.2%), the United Kingdom (8.5–12.5%), and Italy (9%) (Emaneini et al., 2016). Olivier et al. (2008) observed that of the 768 patients colonized with VRE, 4.0% usually developed VRE BSIs due to a related strain; therefore, continuously monitoring trends in the microbiology of BSI pathogens is very important. In general, for both *S. hominis* and *E. faecium* isolated from BSIs, RICU and GICU exhibited more serious antibiotic resistance than other ICUs in our study. In addition, the antibiotic-resistant pathogens of BSIs in Peking University First Hospital were generally more than that in Miyun Teaching Hospital. The phenomenon might result from the complexity of the hospitalized patient’s illness and inappropriate use of antimicrobials in primary hospitals or secondary hospitals as the multidrug-resistant bacteria emerged before the submission into tertiary hospitals which then prescribed more broad-spectrum antibiotics in tertiary hospital. As a predominant Gram-negative bacterium in blood cultures, *K*. *pneumoniae* was highly susceptible to doxycycline, minocycline, and tigecycline in all the ICUs except RICU, and its antibiotic sensitivity to imipenem, meropenem, amikacin, ciprofloxacin, and levofloxacin in the GICU was high or moderate in the PICU, CICU, and GICU, but mild in the SICU and RICU. *K*. *pneumoniae* is a common cause of healthcare- and ICU-acquired infections, and one of the key pathogens from bacterial BSIs (Kern and Rieg, 2020). Increasingly used carbapenem has contributed significantly to the emergence and rapid dissemination of carbapenem-resistant *K. pneumoniae* (CRKP) strains (Landman et al., 2007; Girometti et al., 2014). Moreover, carbapenem-resistant *Enterobacteriaceae* (CRE) [including CRKP, carbapenem-resistant *Pseudomonas aeruginosa* (CRPA), etc] was one of the major antibiotic resistance types in RICU, SICU, and CICU. The multidrug-resistance patterns such as CRE and carbapenem-resistant *A. baumannii* (CRAB) have been increasing in recent years. In the ICUs of our research, *A. baumannii* only exhibited high susceptibility to tigecycline and mild sensitivity to imipenem and meropenem. Its susceptibility to ceftazidime, cefepime, tobramycin, levofloxacin, doxycycline, and minocycline was moderate in the PICU, but mild in the SICU and RICU. Moreover, CRAB was one of the common multidrug-resistance patterns in the PICU. Liu et al. (2020) conducted a prospective multicenter study in China and suggested that carbapenem resistance has a significant impact on mortality for patients with *A. baumannii* complex BSI. *E. coli* was highly susceptible to the tested antibiotics in the CICU, whereas it exhibited moderate or relatively low drug susceptibility to the antibiotics (except doxycycline, minocycline, and tigecycline) in the PICU, SICU, and GICU. In our study, for *K*. *pneumoniae*, *A. baumannii*, and *E. coli* isolated from BSIs, RICU, SICU, and GICU exhibited more serious antibiotic resistance than other ICUs. The phenomenon may result from inappropriate empirical use of antimicrobials for patients with respiratory tract infections or to avoid suspected infection of surgical operations. Furthermore, age is probably a main contributor to the multidrug-resistant bacteria emerged among the geriatric patients undergoing prolonged treatments with broad-spectrum antibiotics.

In this study, the blood cultures were obtained from several different ICUs (including pediatric, adult, or elderly patients) in one urban and one outer suburban hospital. The sample size of this dual-center retrospective study from 2017 to 2020 was relatively large. Therefore, the bacterial distributions and microbial susceptibility patterns analyzed in various ICUs and distinct age groups were relatively solid. However, our research has certain limitations, partly because it was only focused on BSIs from one tertiary hospital and outer suburban hospital in Beijing, which might have led to enrollment bias considering the geographic location.

## Conclusion

The distribution of prevalent microorganisms and their antibiotic susceptibility revealed a high divergence among BSIs in different ICUs from a tertiary hospital and outer suburban hospital. RICU and GICU exhibited more serious antibiotic resistance than other ICUs. Therefore, different antibiotic therapies for various wards and distinct age groups were suggested, further strengthening the importance of active prevention and control strategies for the emergence and spread of highly antibiotic-resistant infections.

## Data Availability Statement

The original contributions presented in the study are included in the article/supplementary material, further inquiries can be directed to the corresponding author/s.

## Ethics Statement

The studies involving human participants were reviewed and approved by the Peking University First Hospital and Miyun Teaching Hospital of Capital Medical University. Written informed consent for participation was not required for this study in accordance with the national legislation and the institutional requirements.

## Author Contributions

ND designed the study, carried out the data collection, drafted the initial manuscript, and reviewed and revised the manuscript. LS carried out the data collection and reviewed and revised the manuscript. CH coordinated the data collection and reviewed the manuscript. HL designed the study and critically reviewed the manuscript for important intellectual content. BC reviewed the manuscript for important intellectual content. All authors have read and approved the manuscript.

## Conflict of Interest

The authors declare that the research was conducted in the absence of any commercial or financial relationships that could be construed as a potential conflict of interest.

## Publisher’s Note

All claims expressed in this article are solely those of the authors and do not necessarily represent those of their affiliated organizations, or those of the publisher, the editors and the reviewers. Any product that may be evaluated in this article, or claim that may be made by its manufacturer, is not guaranteed or endorsed by the publisher.

## Abbreviations

BSI: bloodstream infection
ICUs: intensive care units
PICU: pediatric ICU
SICU: surgical ICU
CICU: cardiac ICU
RICU: respiratory ICU
GICU: geriatric ICU
ANRESIS: antibiotic resistance surveillance system
SOP: standard operation procedure
K–B: Kirby–Bauer
CLSI: Clinical Laboratory Standards Institute
ESBL: extended-spectrum β -lactamase
CRE: carbapenem-resistant *Enterobacteriaceae*
CRAB: carbapenem-resistant *Acinetobacter baumannii*
MRSA: methicillin-resistant *Staphylococcus aureus*
MRCNS: methicillin-resistant coagulase-negative *Staphylococcus*
VRE: vancomycin-resistant *Enterococcus*
SIRS: systemic inflammatory response syndrome
CoNS: coagulase-negative staphylococci.
